# Supplementary material for: Silica-grafted ionic liquids for revealing the respective charging behaviors of cations and anions in supercapacitors
Source: Nat Commun. 2017 Dec 19;8:2188. doi: 10.1038/s41467-017-02152-5 (PMC5736757; doi:10.1038/s41467-017-02152-5)
Supplement: Supplementary file 1 — Supplementary Information [file 41467_2017_2152_MOESM1_ESM.doc]

**Supplementary Information**


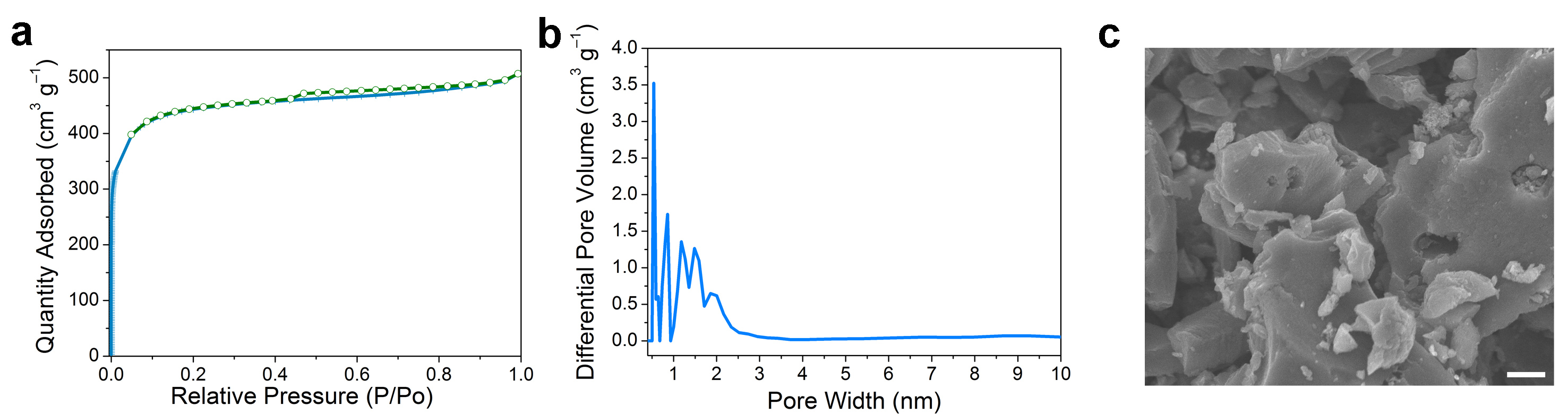


**Supplementary Figure 1 | Characterization of activated carbon YP-50F. a**, Nitrogen adsorption-desorption isotherm. **b**, Pore size distribution. **c**, A typical scanning electron microscope (SEM) image. Scale bars, 1 µm.


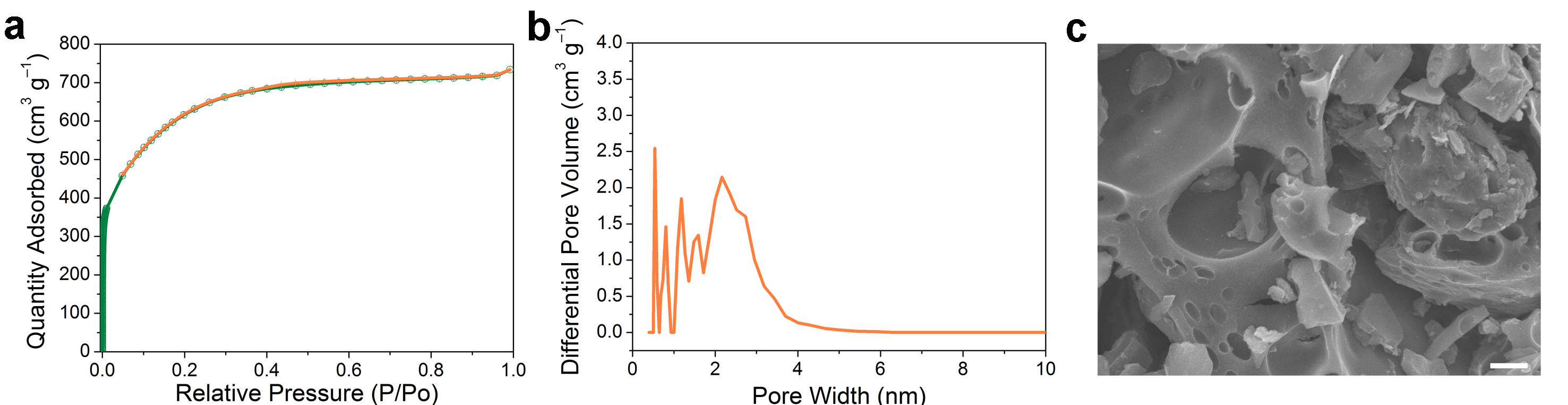


**Supplementary Figure 2 | Characterization of sodium alginate-derived activated carbon (SADC). a**, Nitrogen adsorption-desorption isotherm. **b**, Pore size distribution. **c**, A typical scanning electron microscope (SEM) image. Scale bars, 1 µm.

**
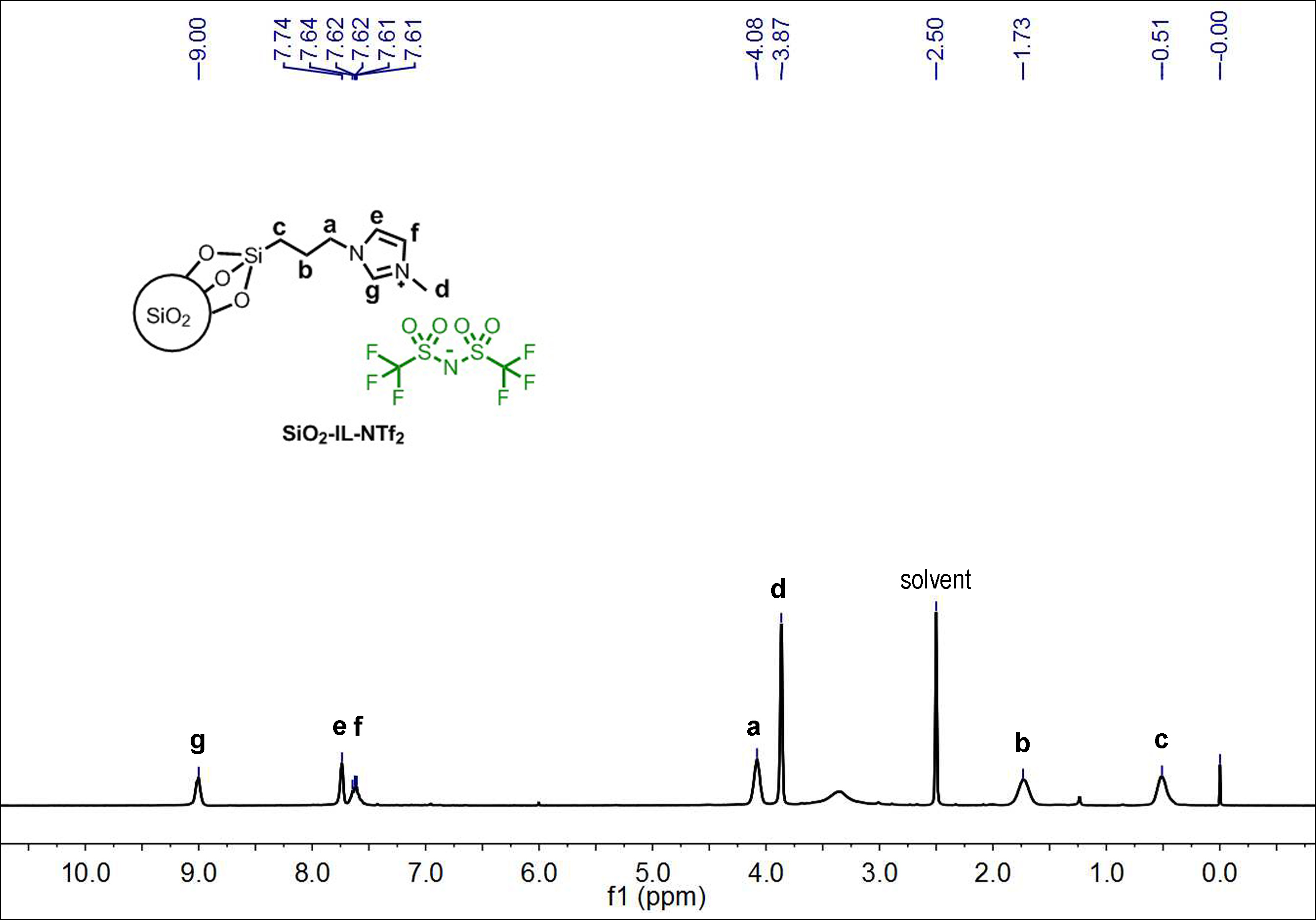
**

**Supplementary Figure 3 |** The ^1^H NMR spectrum of SiO_2_-IL-NTf_2_.

**
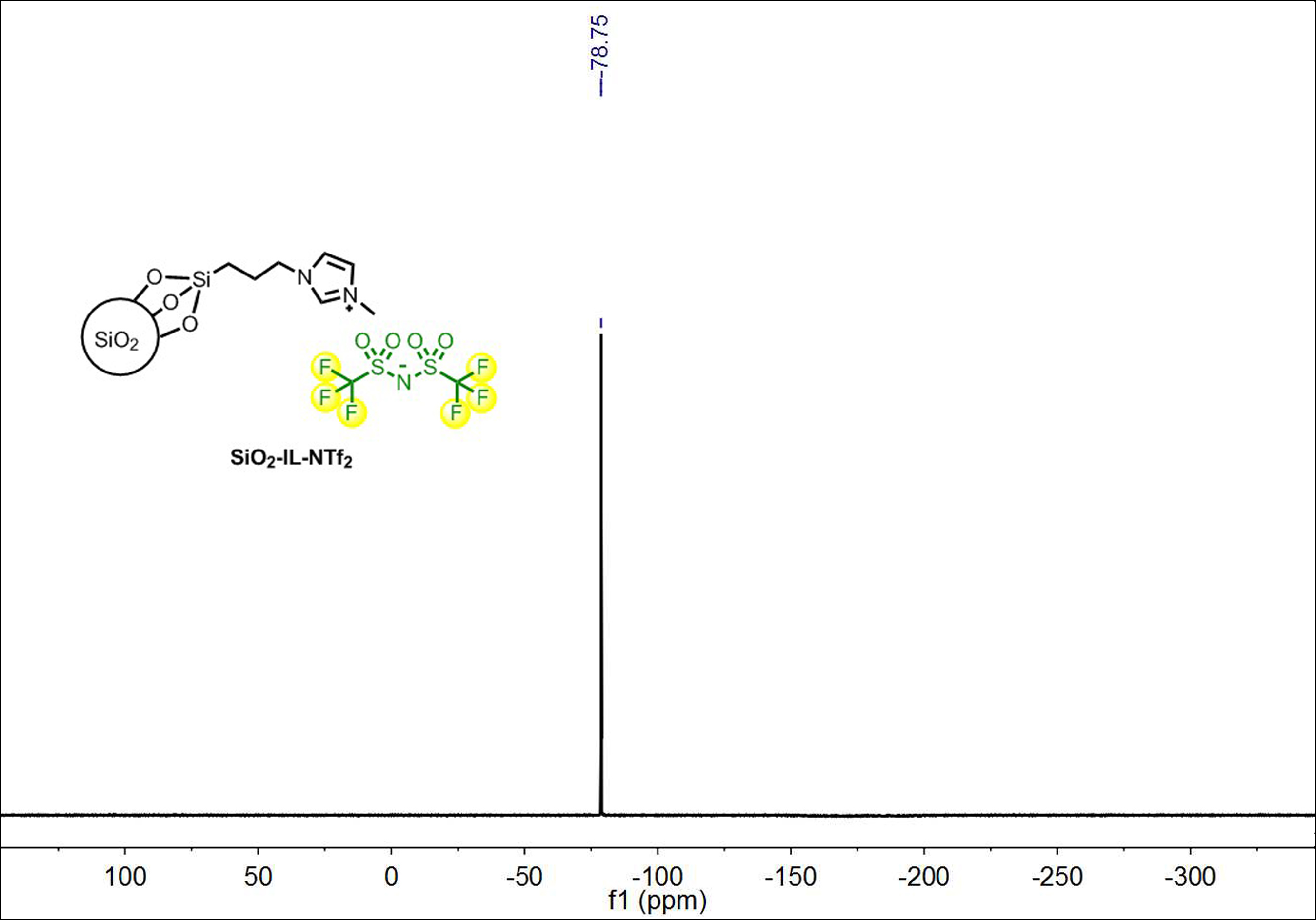
**

**Supplementary Figure 4 |** The ^19^F NMR spectrum of SiO_2_-IL-NTf_2_.

**
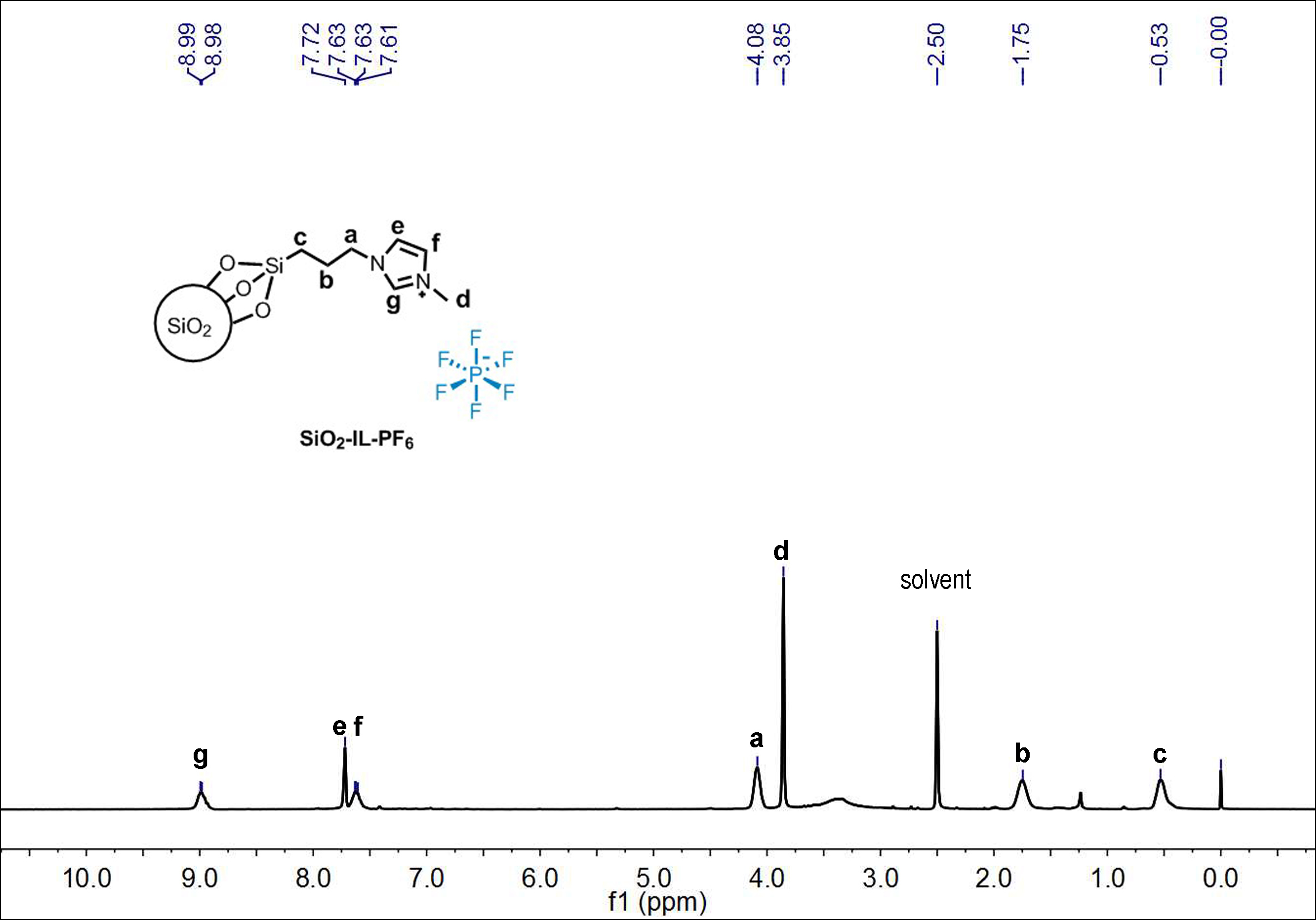
**

**Supplementary Figure 5 |** The ^1^H NMR spectrum of SiO_2_-IL-PF_6_.

**
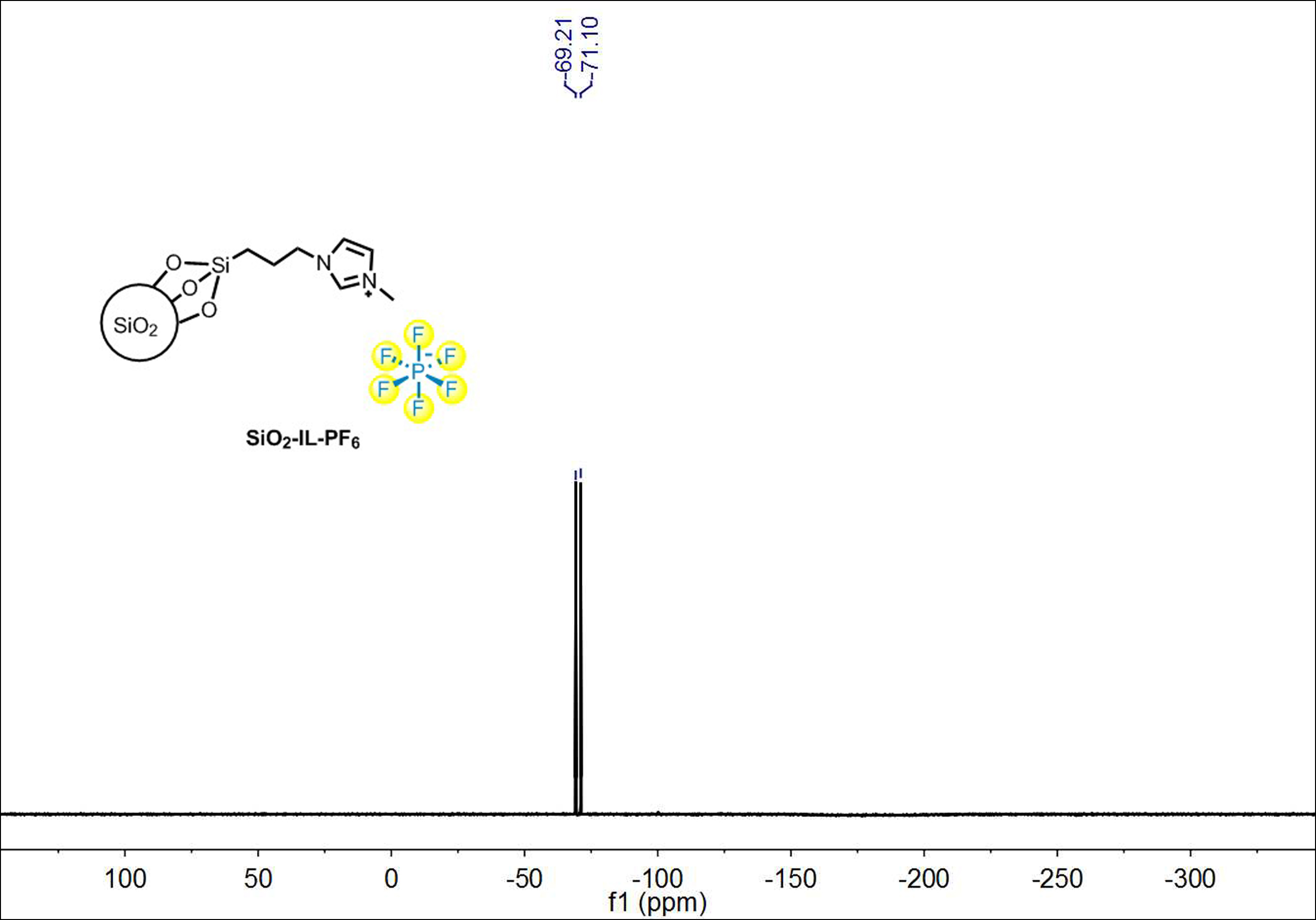
**

**Supplementary Figure 6 |** The ^19^F NMR spectrum of SiO_2_-IL-PF_6_.

**
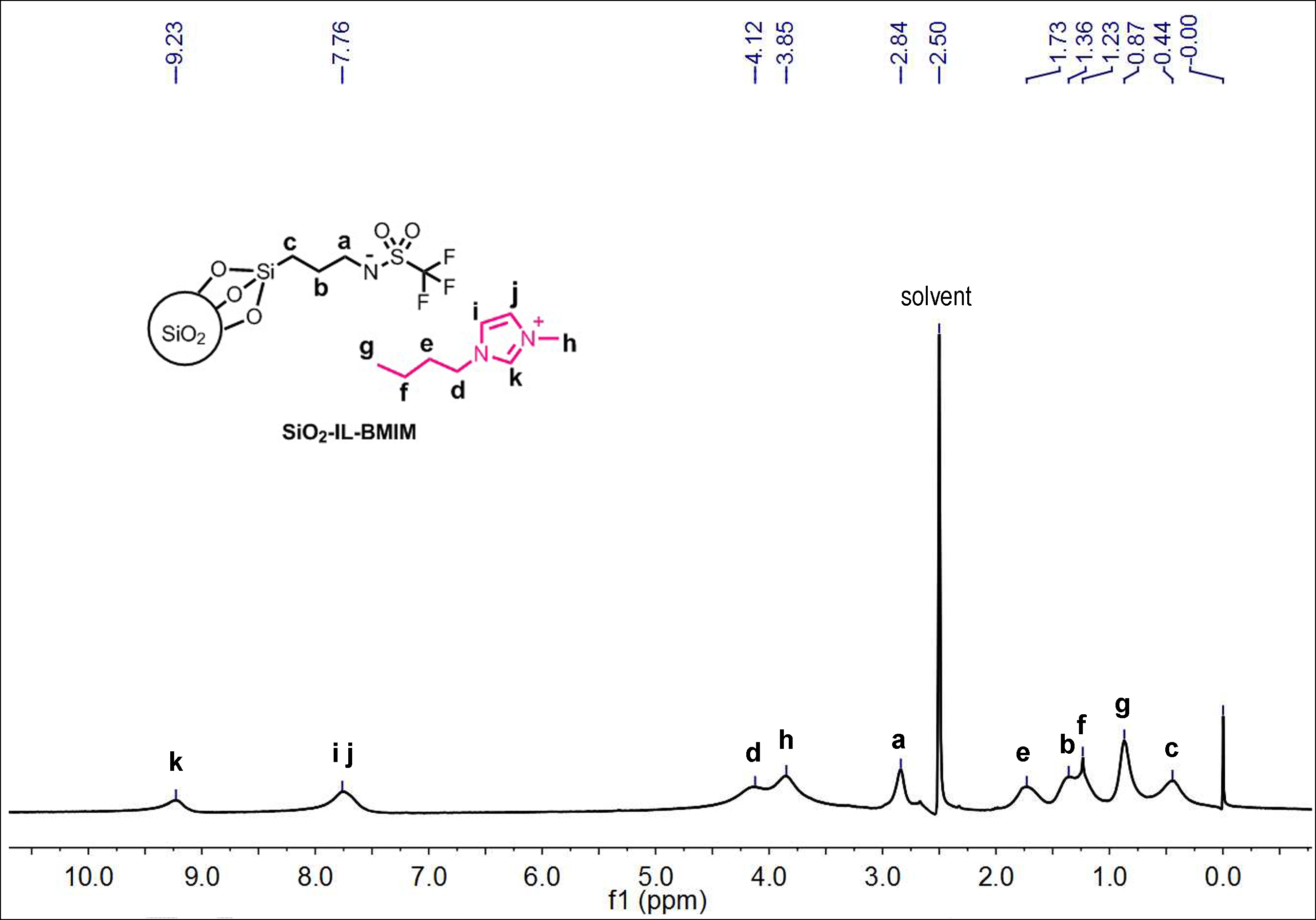
**

**Supplementary Figure 7 |** The ^1^H NMR spectrum of SiO_2_-IL-BMIM.

**
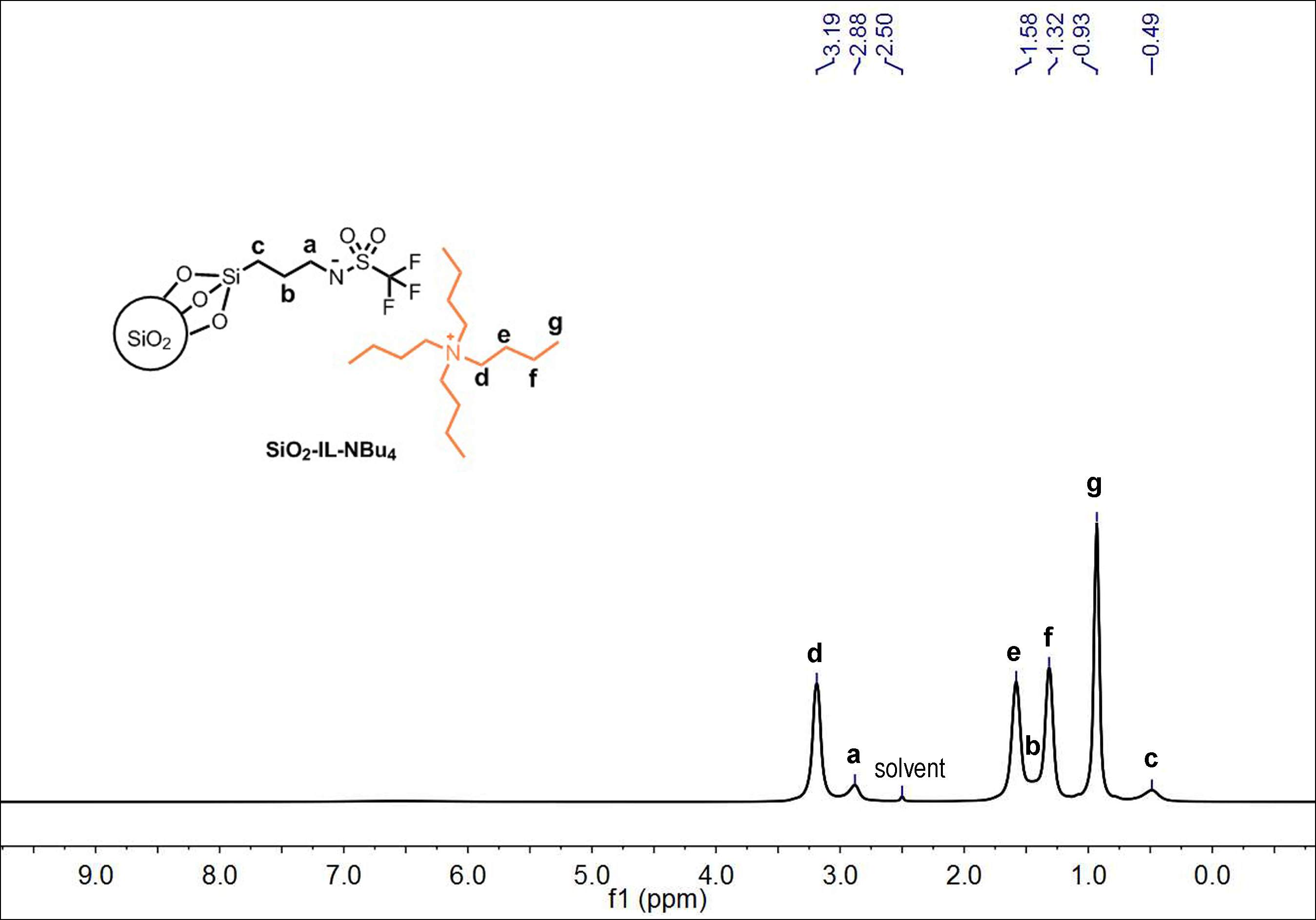
**

**Supplementary Figure 8 |** The ^1^H NMR spectrum of SiO_2_-IL-NBu_4_.

**
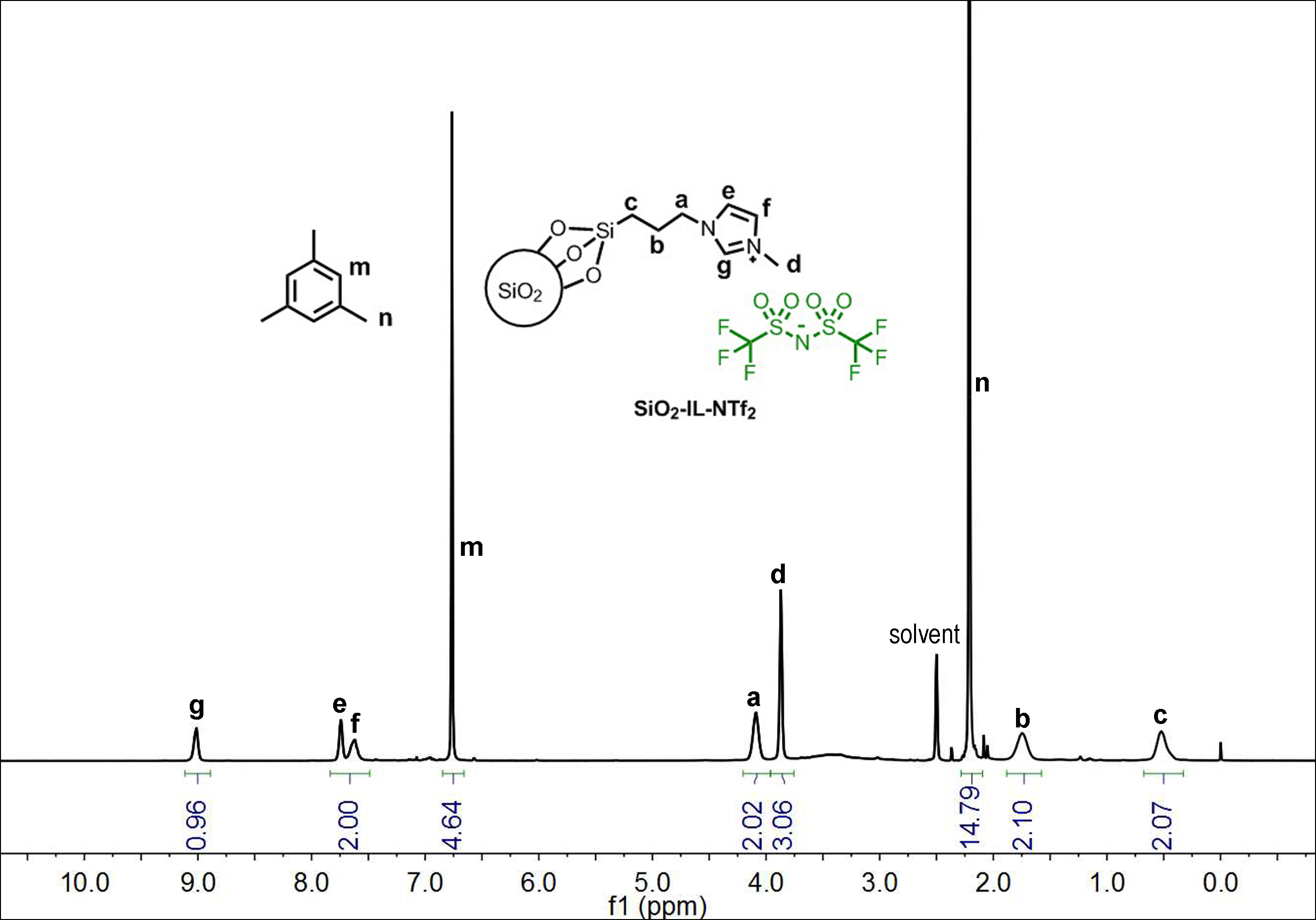
**

**Supplementary Figure 9 |** The ^1^H NMR spectrum of SiO_2_-IL-NTf_2_ and mesitylene. The ion concentration of SiO_2_-IL-NTf_2_ (**1**) was 0.93 mmol g^−1^. $n=\frac{1}{4.64/3}\cdot\frac{0.0721}{0.05}=0.93$.

**
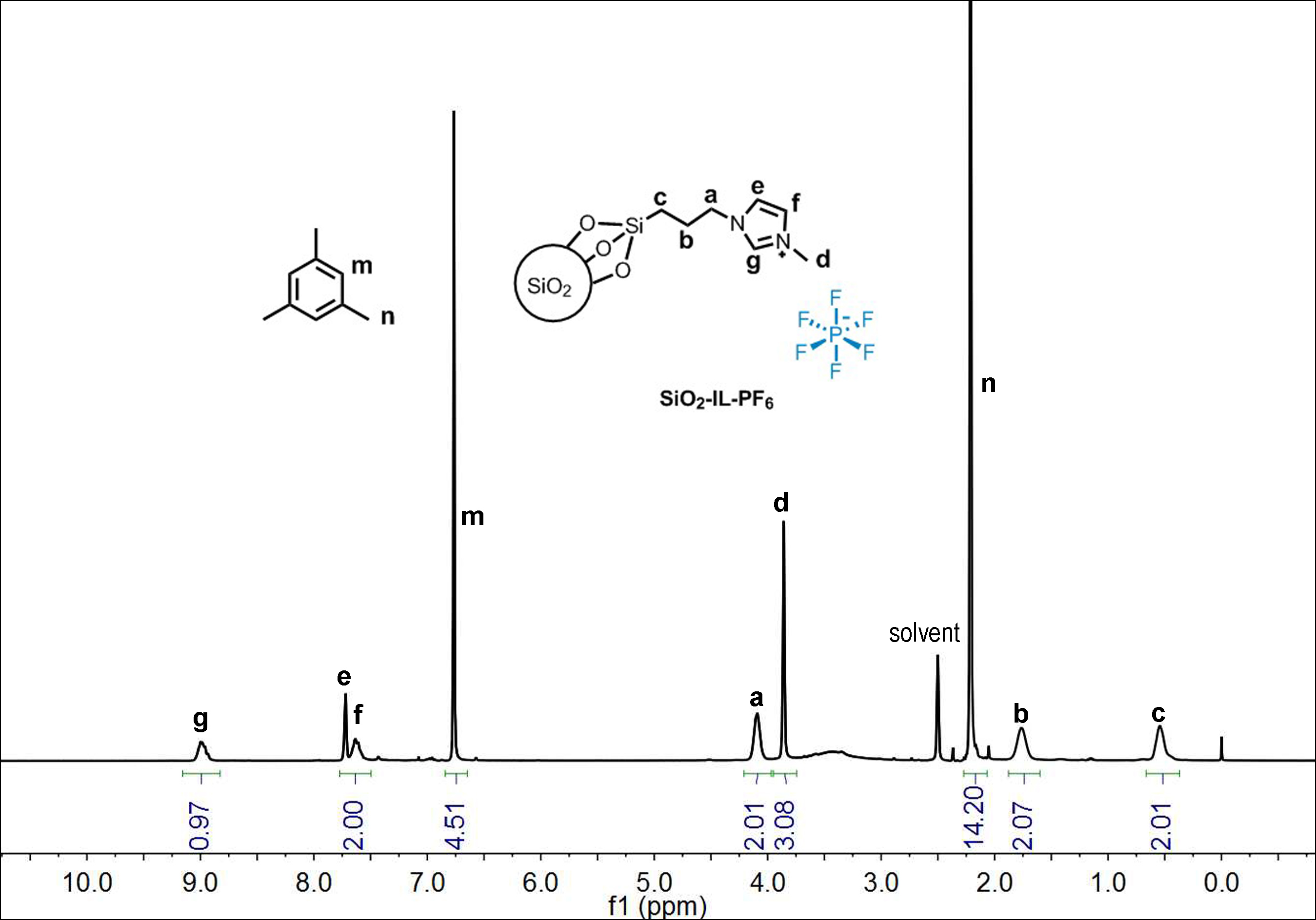
**

**Supplementary Figure 10 |** The ^1^H NMR spectrum of SiO_2_-IL-PF_6_ and mesitylene. The ion concentration of SiO_2_-IL-PF_6_ (**2**) was 0.95 mmol g^−1^. $n=\frac{1}{4.51/3}\cdot\frac{0.0721}{0.05}=0.95$.

**
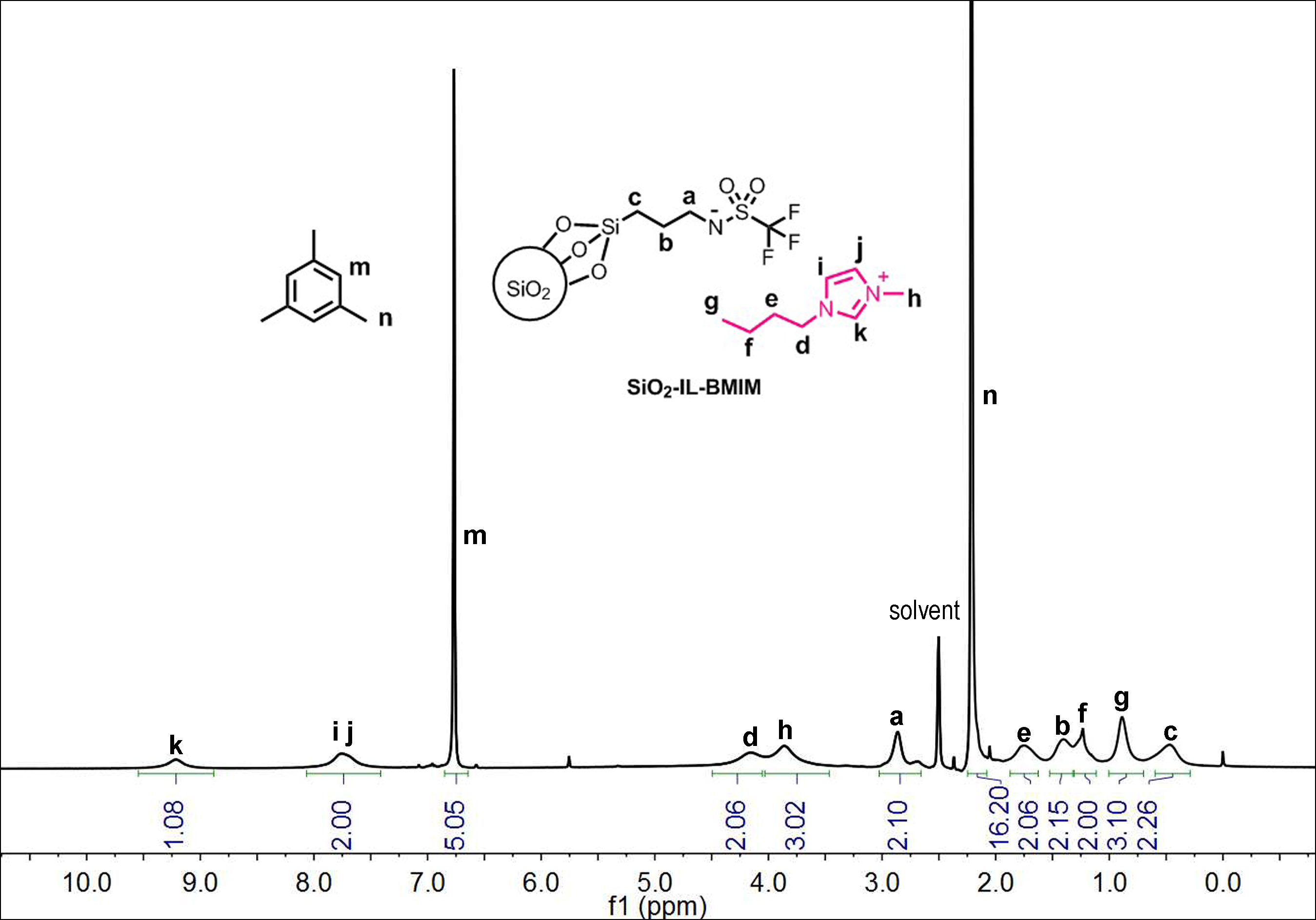
**

**Supplementary Figure 11** **|** The ^1^H NMR spectrum of SiO_2_-IL-BMIM and mesitylene. The ion concentration of SiO_2_-IL-BMIM (**3**) was 0.86 mmol g^−1^. $n=\frac{1}{5.05/3}\cdot\frac{0.0721}{0.05}=0.86$

**
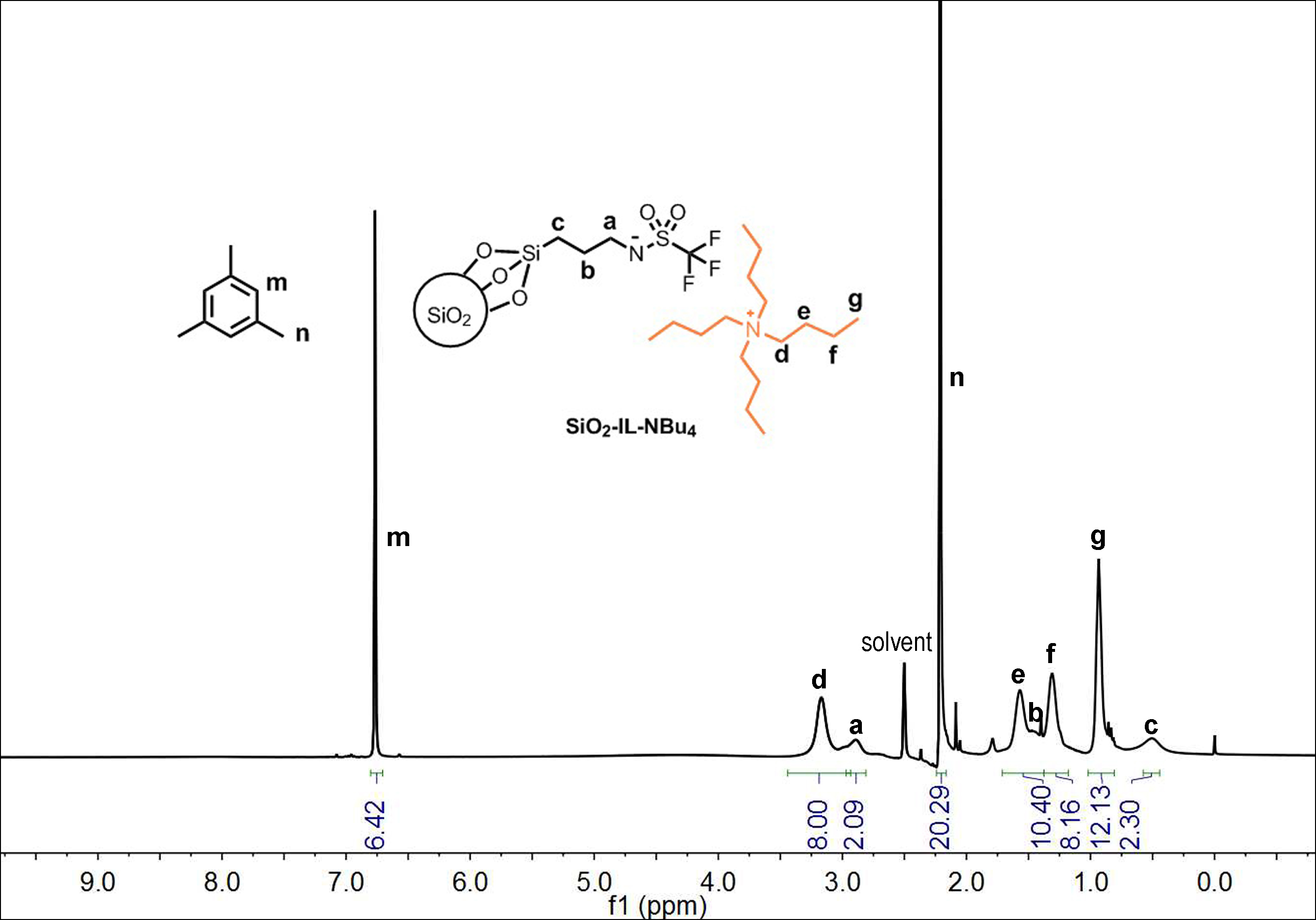
**

**Supplementary Figure 12 |** The ^1^H NMR spectrum of SiO_2_-IL-NBu_4_ and mesitylene. The ion concentration of SiO_2_-IL-NBu_4_ (**4**) was 0.67 mmol g^−1^. $n=\frac{1}{6.42/3}\cdot\frac{0.0721}{0.05}=0.67$

**
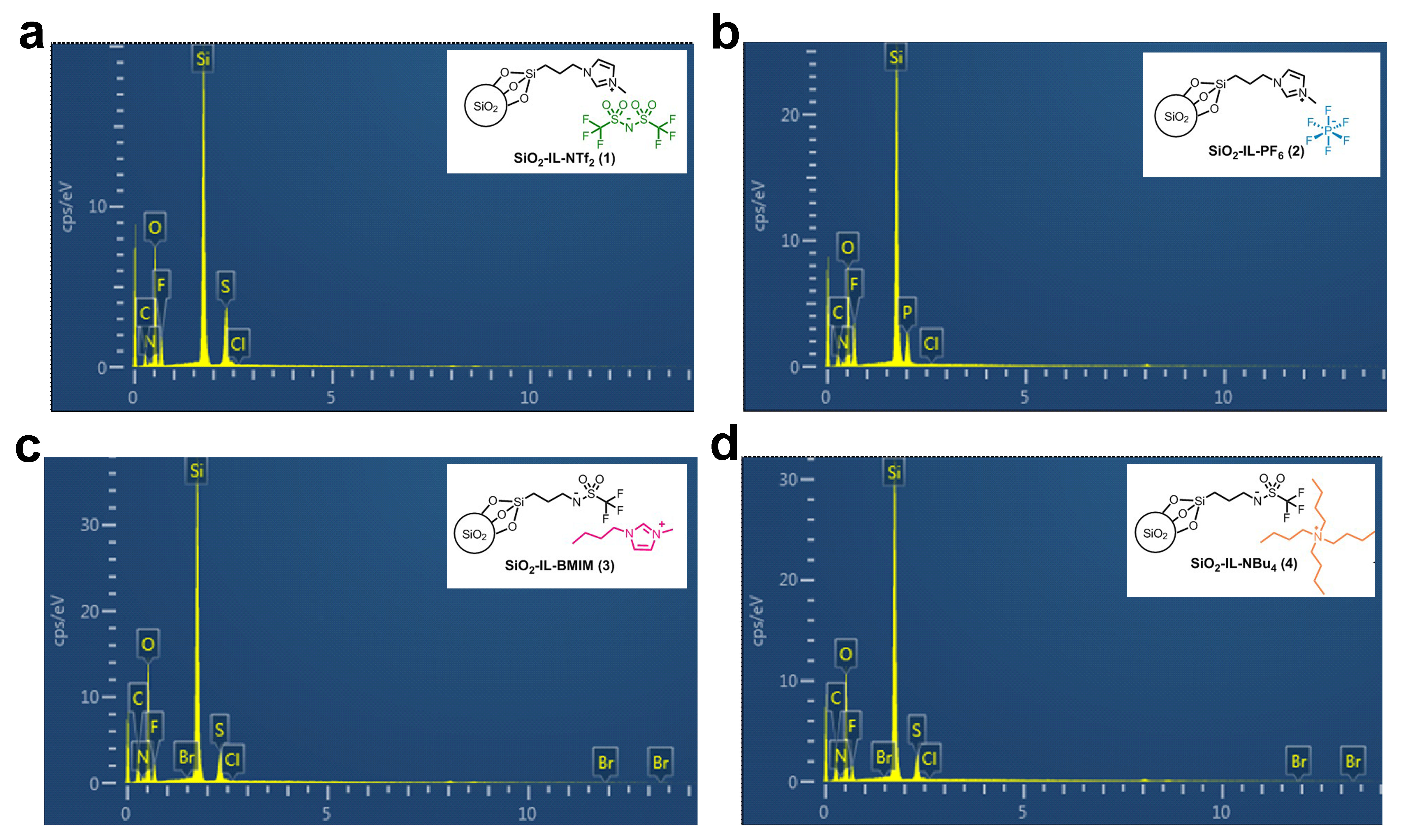
**

**Supplementary Figure 13 |** Energy-dispersive X-ray spectroscopy (EDS) elemental analysis of (**a**) SiO_2_-IL-NTf_2_, (**b**) SiO_2_-IL-PF_6_, (**c**) SiO_2_-IL-BMIM, (**d**) SiO_2_-IL-NBu_4_. The data indicated that the absence of Cl or Br element in the synthesized four samples.


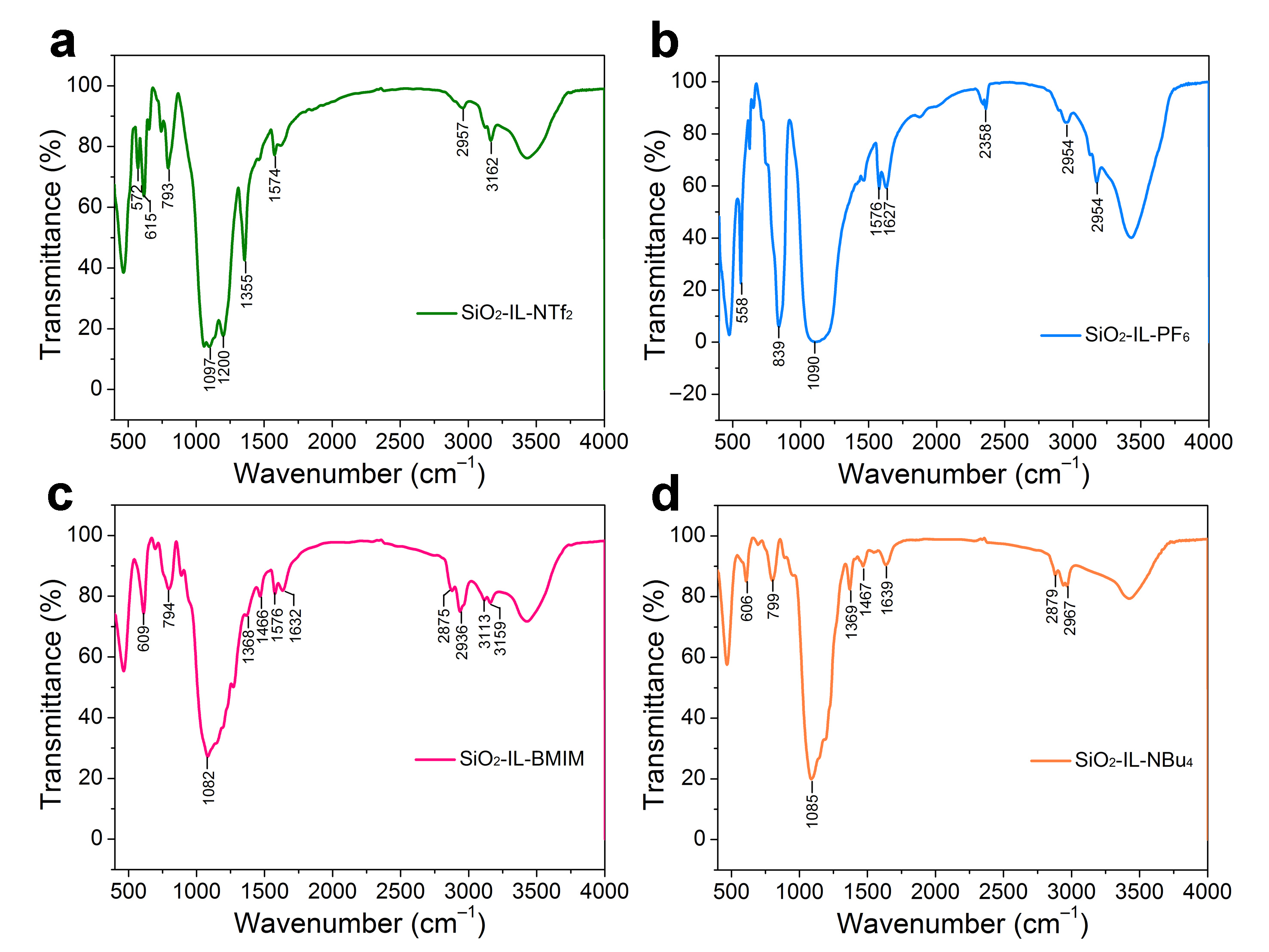


**Supplementary Figure 14 |** Infrared (IR) spectra of (**a**) SiO_2_-IL-NTf_2_, (**b**) SiO_2_-IL-PF_6_, (**c**) SiO_2_-IL-BMIM, (**d**) SiO_2_-IL-NBu_4_. The IR spectra of all SiO_2_-grafted ILs exhibit broad and strong frequencies around 1080 cm^−1^ belonging to *ν*(Si-O). The high-frequency range of 2800~3000 cm^−1^ belongs to *ν*(C-H) of the alkyl chains and the range of 3000~3200 cm^−1^ belongs to *ν*(C-H) of the imidazolium ring. The range of 1350~1550 cm^−1^ belongs to *δ*(C-H) of the alkyl chains and the range of 1550~1650 cm^−1^ belongs to *ν*(C-N), *ν*(C-C) and *δ*(C-H) of the imidazolium ring. The *δ*(C-F), *ν*(C-N) and *ν*(C-N) of NTf_2_^−^ are observed at 572, 793 and 1355 cm^−1^, respectively. The *δ*(P-F), *ν*(P-F) of PF_6_^−^ are observed at 558 and 839 cm^−1^, respectively. Here, *ν* represents stretching mode, *δ* represents bending mode^3^.


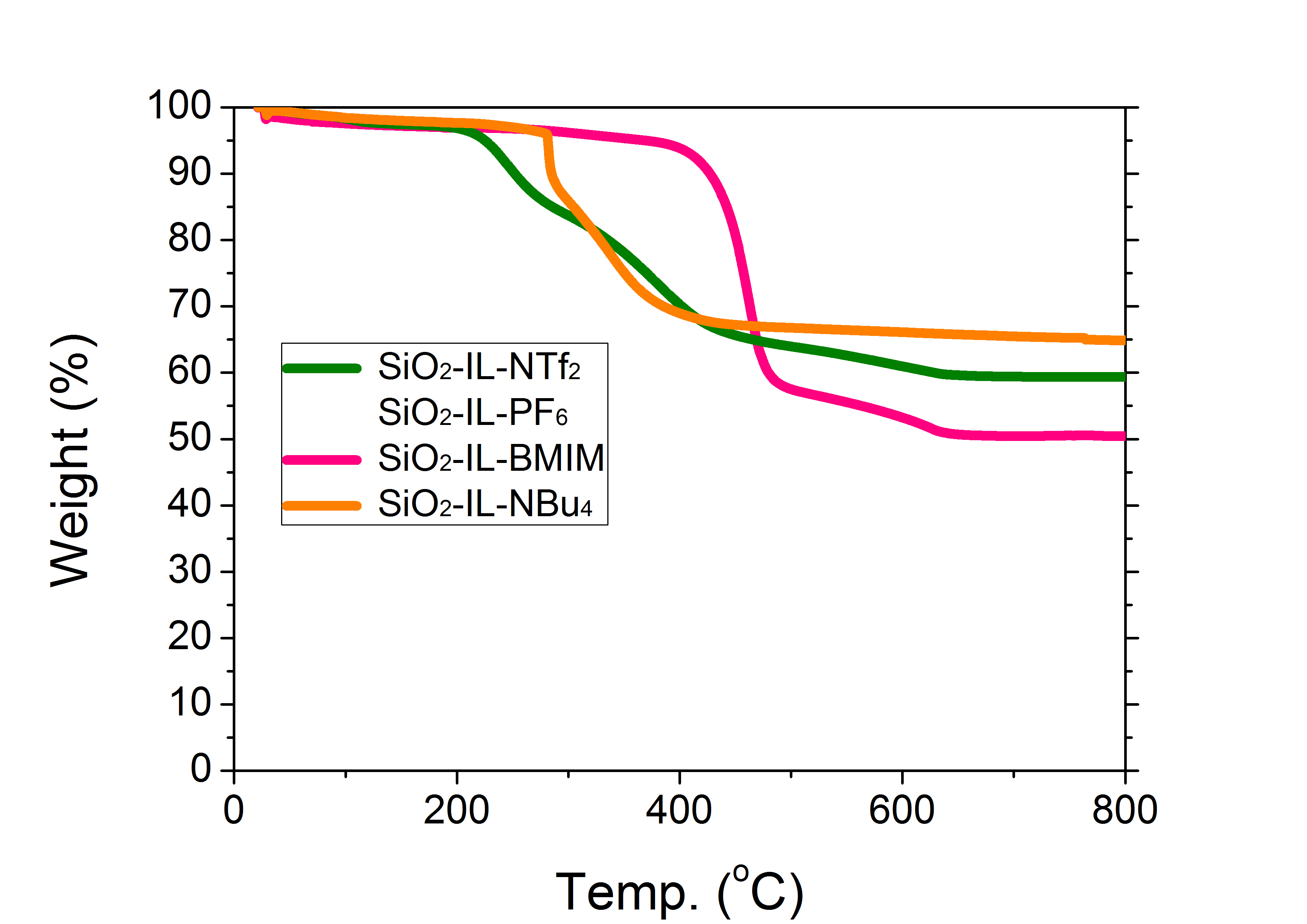


**Supplementary Figure 15 |** Thermogravimetric analysis (TGA) curves of (**a**) SiO_2_-IL-NTf_2_, (**b**) SiO_2_-IL-PF_6_, (**c**) SiO_2_-IL-BMIM, (**d**) SiO_2_-IL-NBu_4_ under air atmosphere.


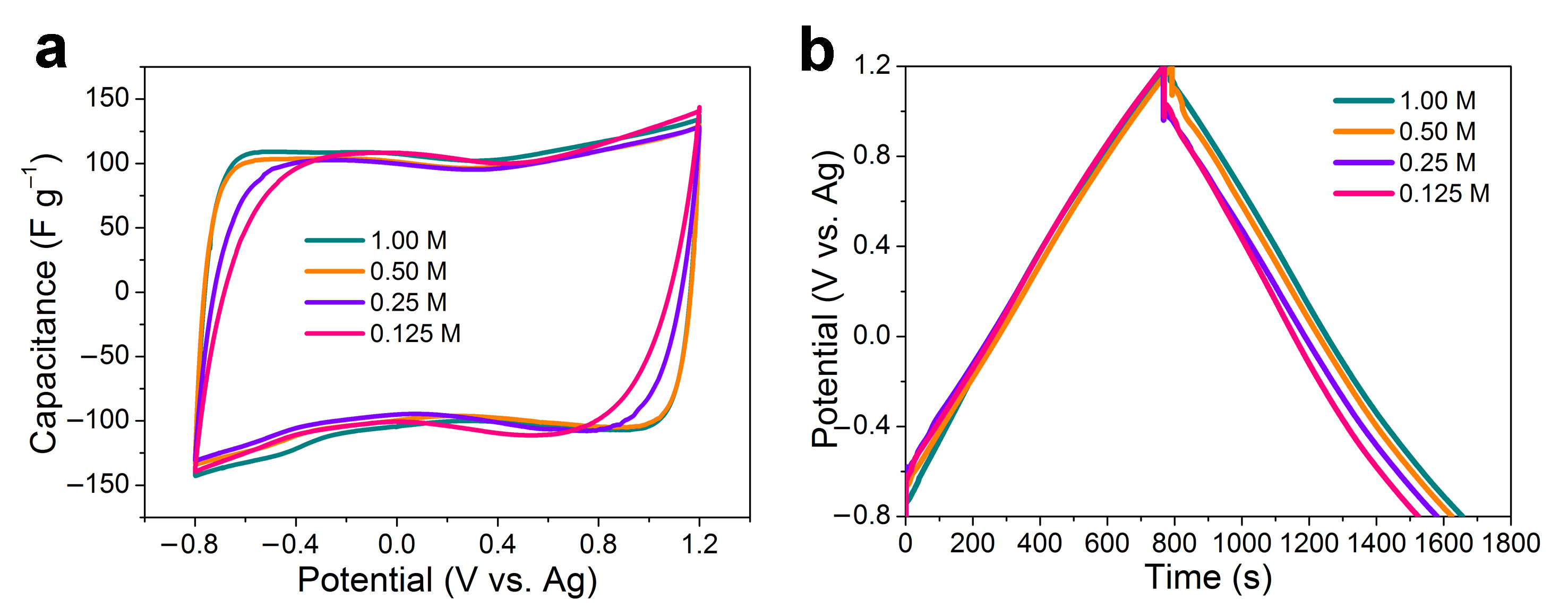


**Supplementary Figure 16 |** **Effect of the concentration of BMIM-NTf_2_/PC on the electrochemical performance of YP-50F electrode.** The cyclic voltammetry curves with a scan rate of 5 mV s^−1^ (**a**) and the galvanostatic charge/discharge curves with a current density of 0.25 A g^−1^ (**b**) at an operating potential window of −0.8 to 1.2 V/ref.


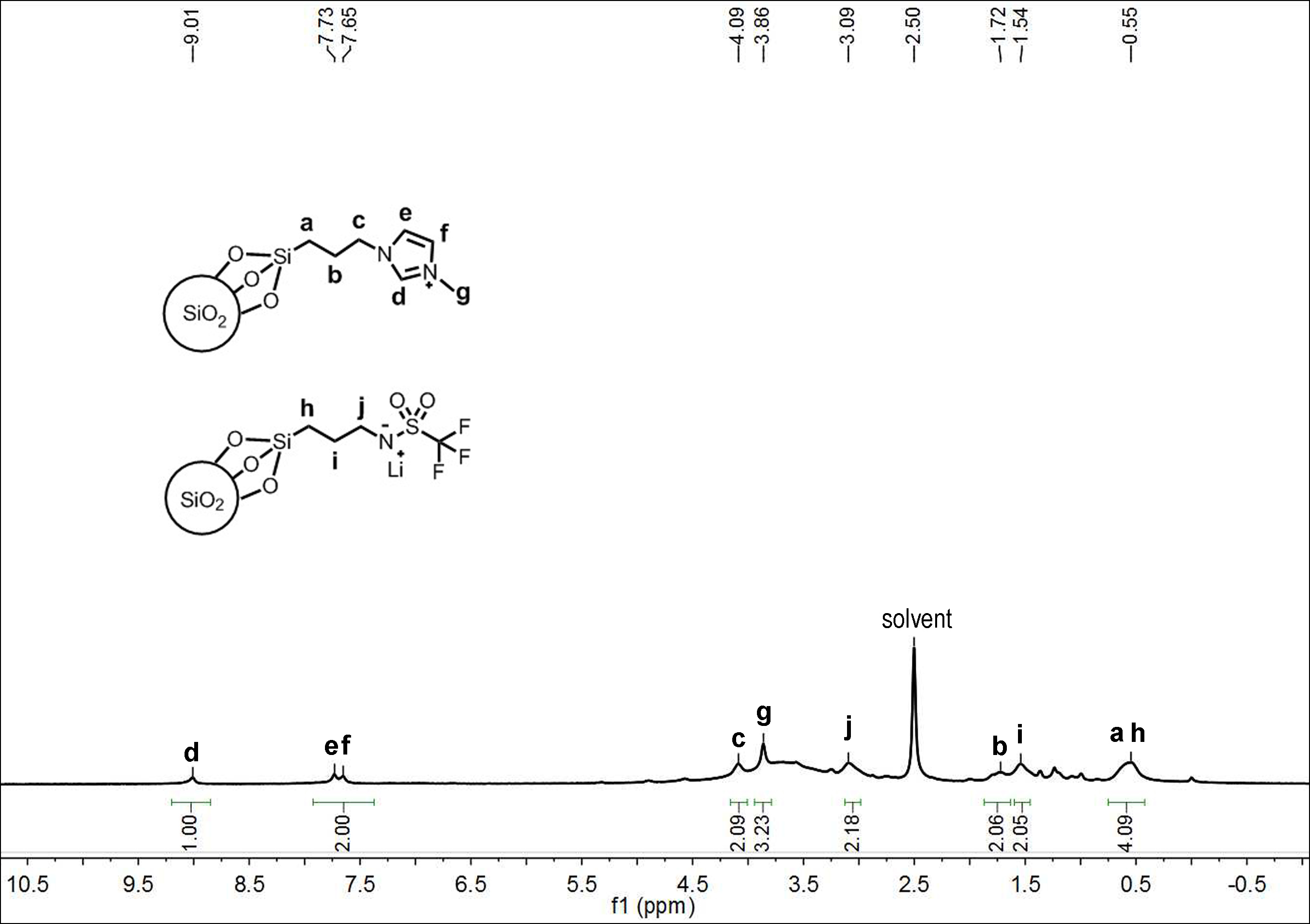


**Supplementary Figure 17 |** The ^1^H NMR spectrum of SiO_2_-MIM–SiO_2_-NTf.


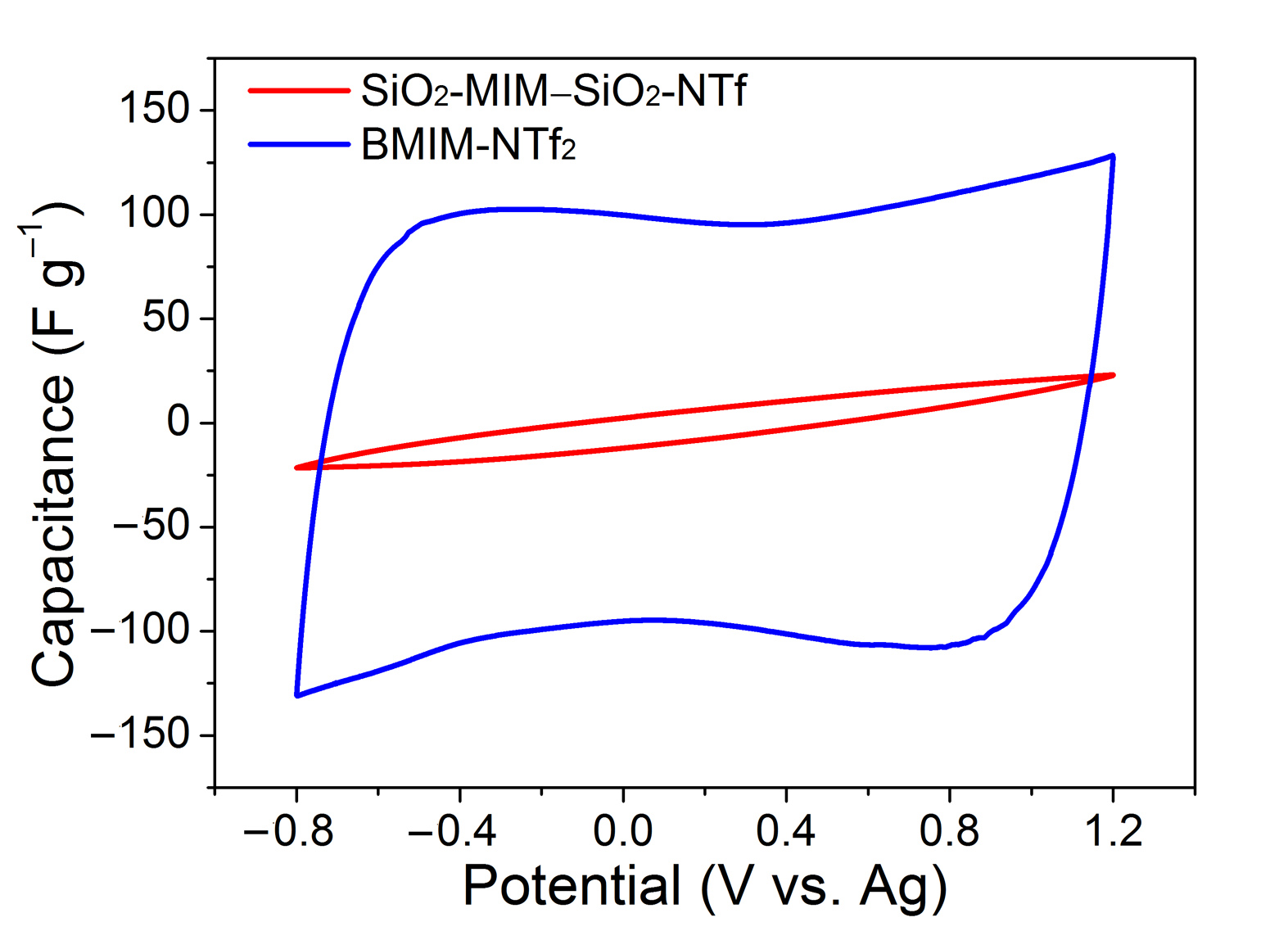


**Supplementary Figure 18 |** Comparison of the cyclic voltammetry curves of YP-50F electrode in SiO_2_-MIM–SiO_2_-NTf/PC and in BMIM-NTf_2_/PC electrolyte within the operating potential window of −0.8~1.2 V with the same scan rate of 5 mV s^−1^.


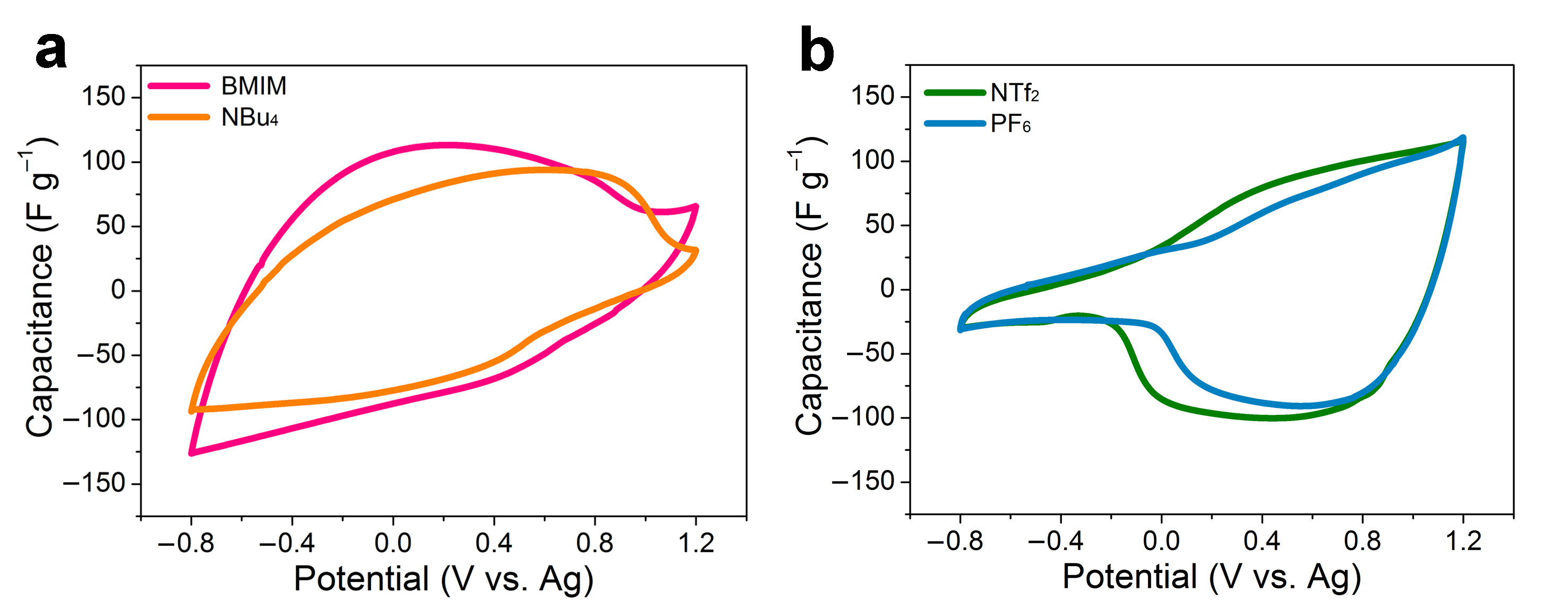


**Supplementary Figure 19 |** Cyclic voltammetry curves of YP-50F electrode in four SiO_2_-grafted ILs with the same concentration of 0.25 M in PC. (**a**) SiO_2_-IL-BMIM and SiO_2_-IL-NBu_4_. (**b**) SiO_2_-IL-NTf_2_ and SiO_2_-IL-PF_6_. The operating potential window was −0.8 to 1.2 V/ref and the scan rate was 5 mV s^−1^. The comparisons indicated that the capacitance contribution of BMIM^+^ was larger than that of NBu_4_^+^ and the capacitance contribution of NTf_2_^−^ was also larger than that of PF_6_^−^.

**
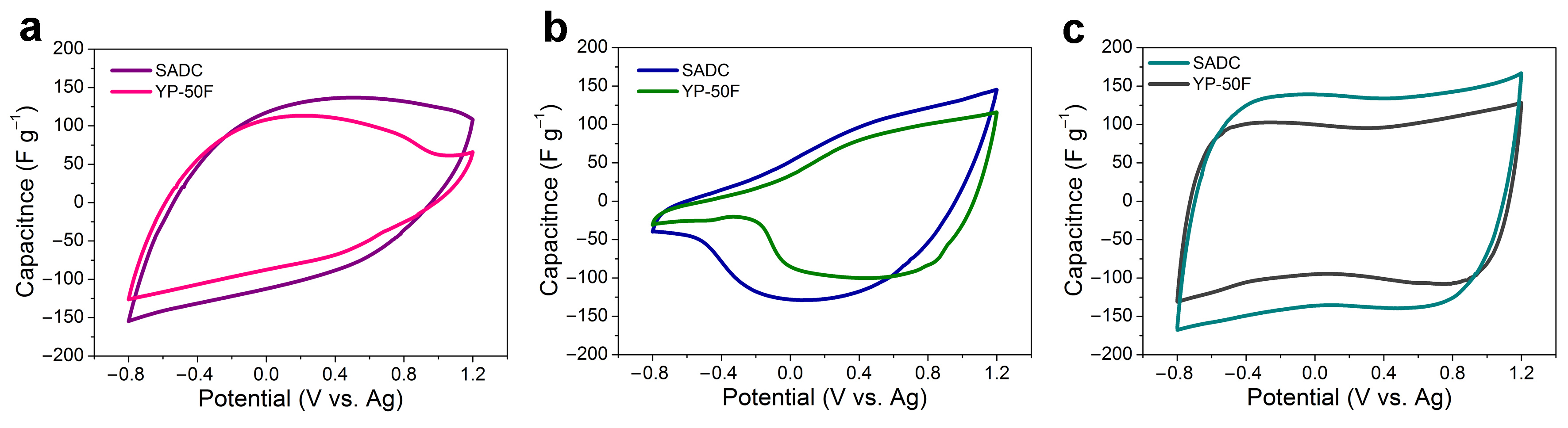
**

**Supplementary Figure 20 |** Cyclic voltammetry curves of YP-50F and SADC electrodes in different ionic liquids with the same concentration of 0.25 M in PC. (**a**) SiO_2_-IL-BMIM. (**b**) SiO_2_-IL-NTf_2_. (**c**) conventional BMIM-NTf_2_. The operating potential window was −0.8 to 1.2 V/ref and the scan rate was 5 mV s^−1^. The comparison indicated that SADC possessed higher capacitance than YP-50F in BMIM-NTf_2_ electrolyte system.


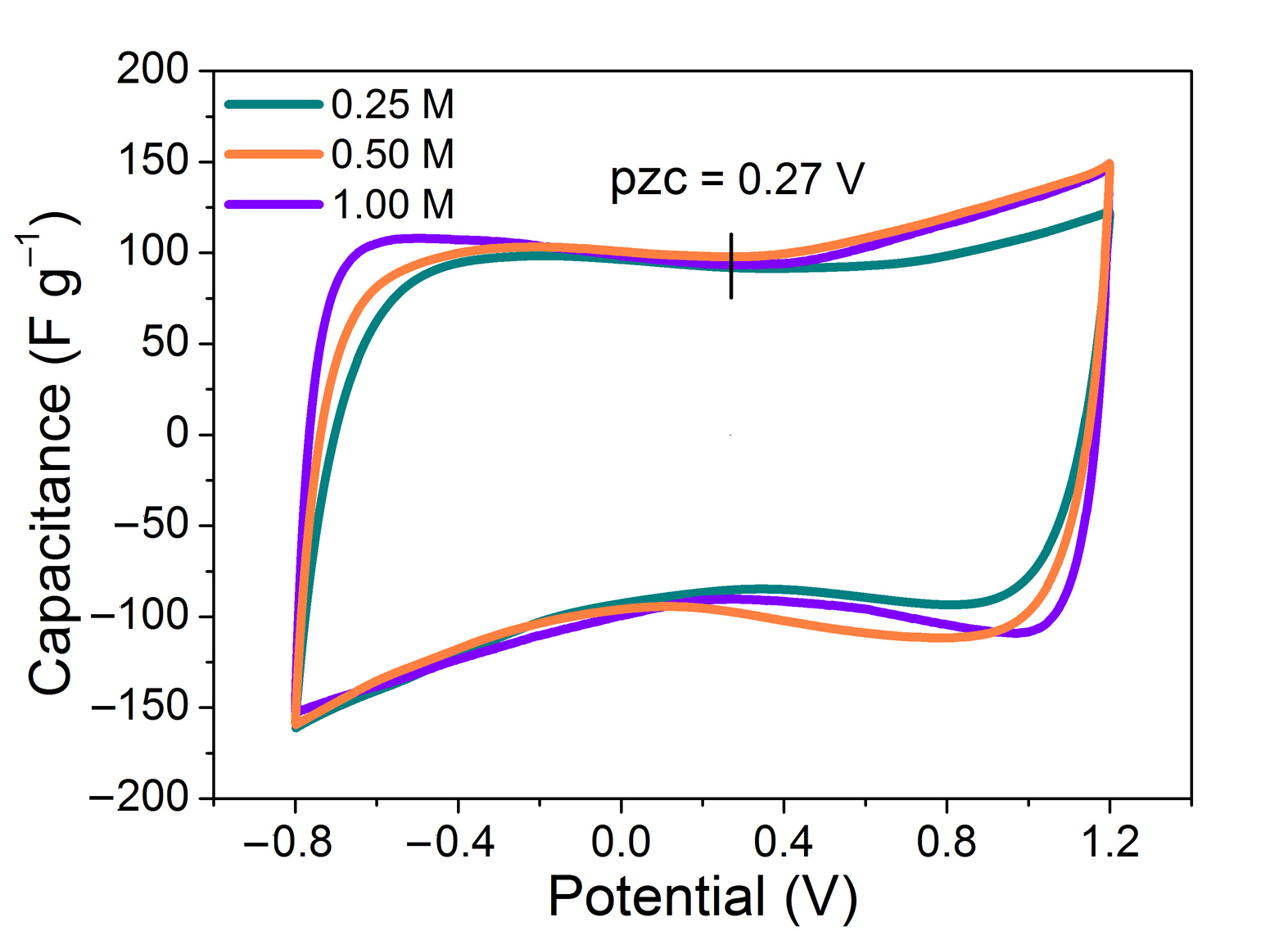


**Supplementary Figure 21 |** Cyclic voltammetry curves of the YP-50F working electrode in BMIM-NTf_2_/PC electrolytes with concentrations of 1.00, 0.50 and 0.25 M at an operating potential window of −0.8 to 1.2 V/ref at a scan rate of 5 mV s^−1^.

**
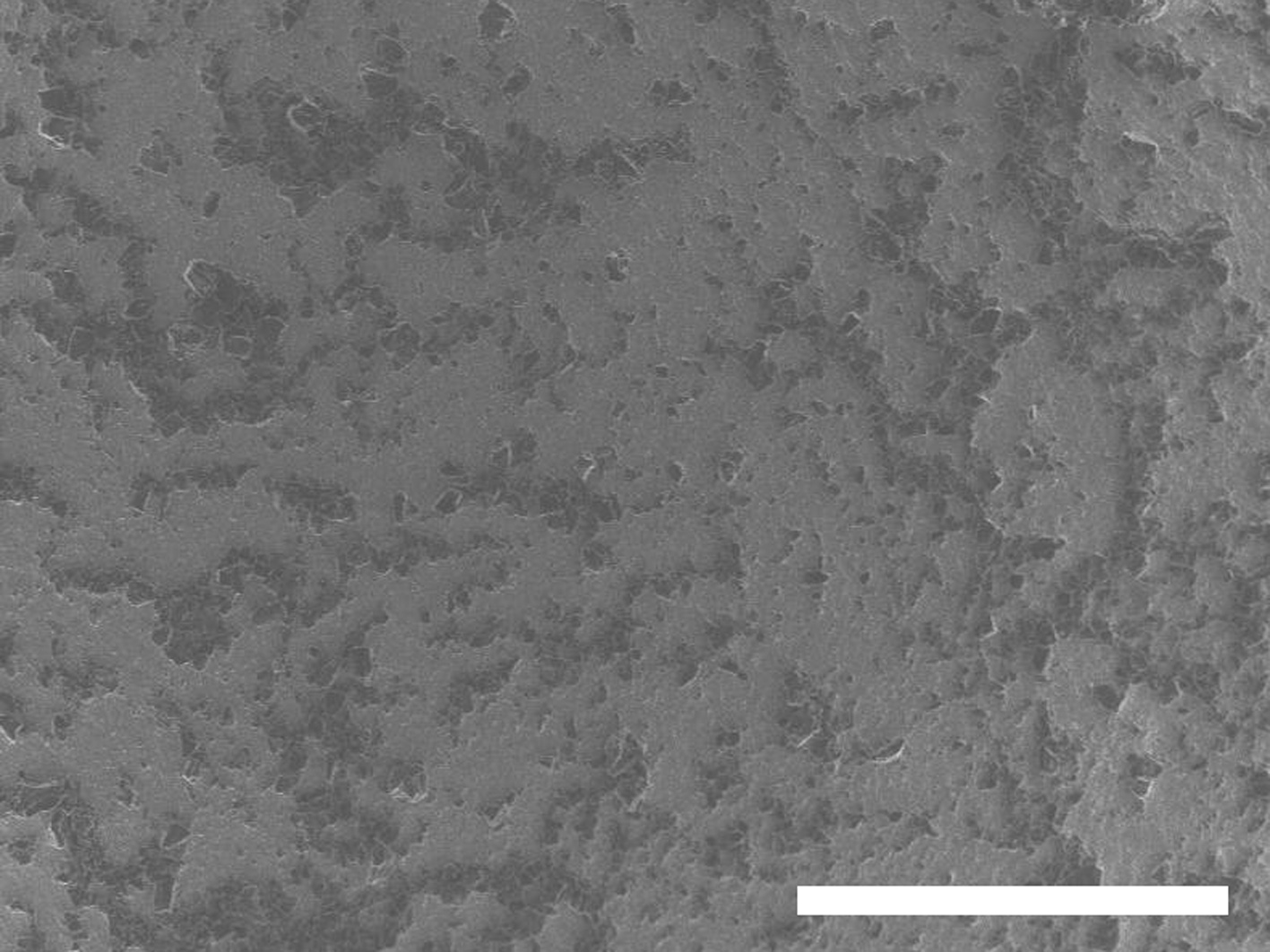
**

**Supplementary Figure 22**| Scanning electron microscope (SEM) images of YP-50F coated on the quartz crystal surface. Scale bars, 1 µm.


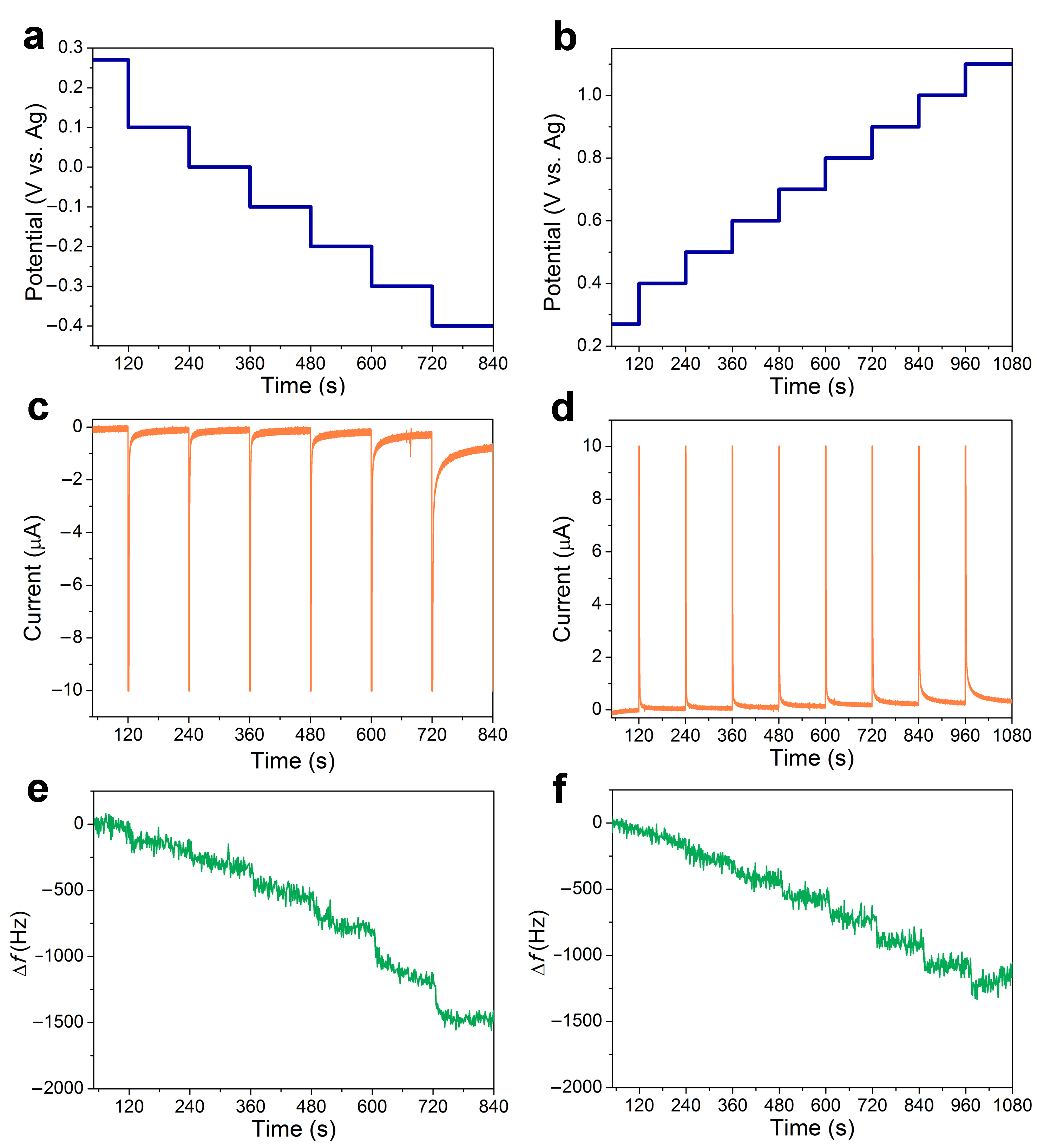


**Supplementary Figure 23 | Chronoamperometry test of the YP-50F-coated quartz electrode in 0.25 M BMIM-NTf_2_ in PC.** The electrode was negatively polarized (**a**) and positively polarized (**b**) in a step of 0.1 V from pzc (0.27 V/ref) and hold at each potential for 120 s. The corresponding current response for the negative polarization (**c**) and the positive polarization (**d**). The corresponding quartz resonance frequency for the negative polarization (**e**) and the positive polarization (**f**).


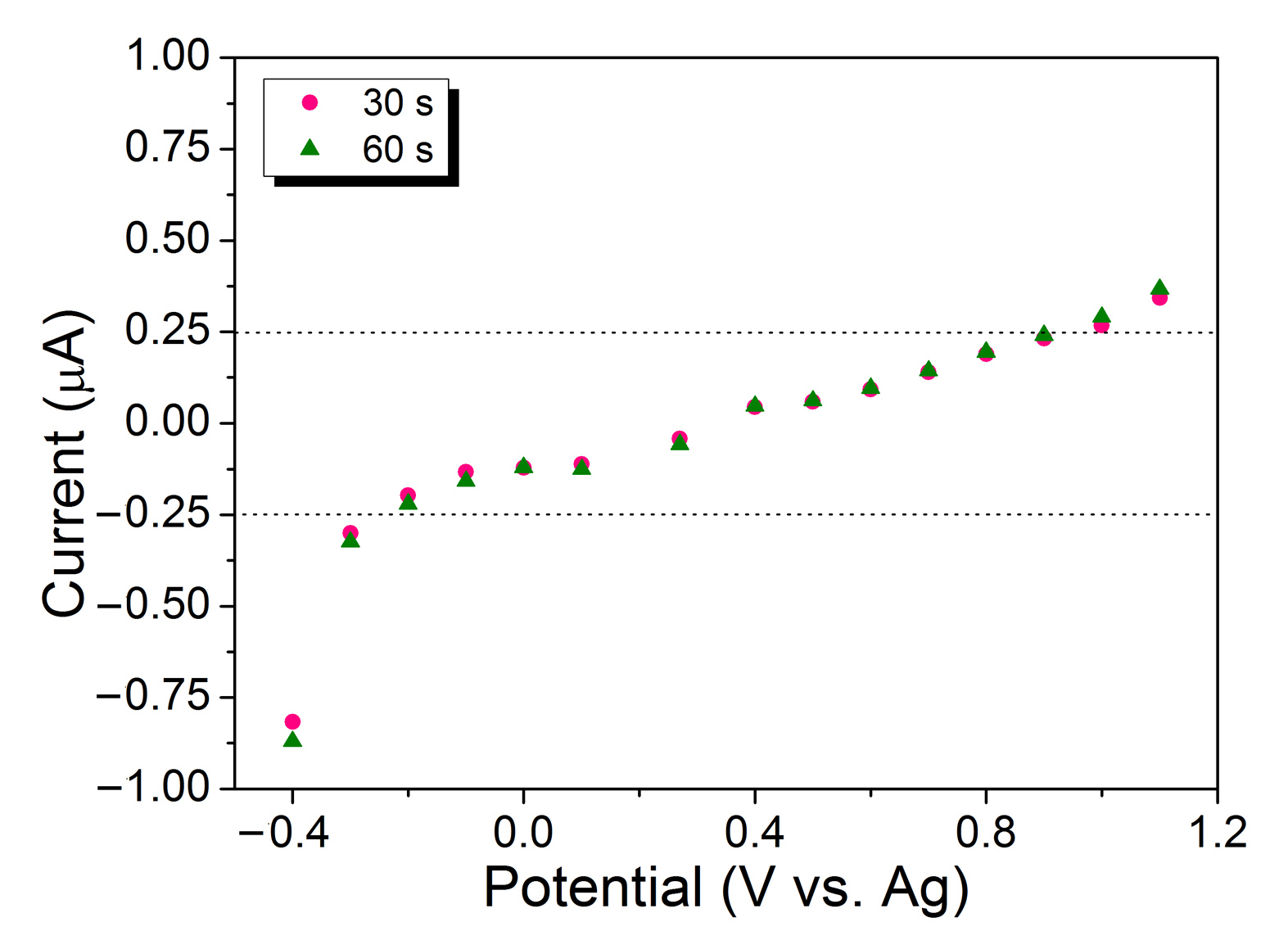


**Supplementary Figure 24 |** The average current of the last 30 or 60 seconds of each polarized potential in Supplementary Figure 23. This figure could give a clearer presentation why the operating potential window in this work was selected to be −0.2 to 0.9 V/ref.


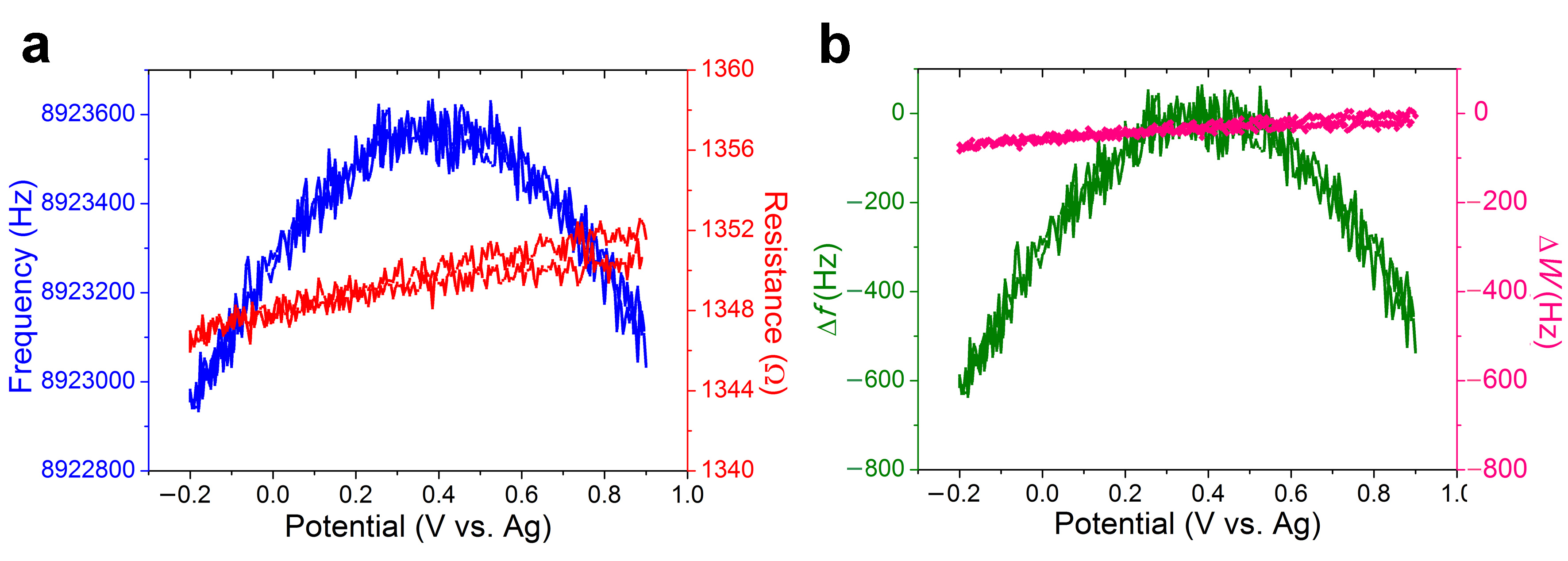


**Supplementary Figure 25** | The original data of resonance frequency and resonance resistance vs potential (**a**), and the related resonance frequency change Δ*f* and resonance width change Δ*W* vs potential (**b**) obtained for YP-50F-coated quartz electrode in BMIM-NTf_2_/PC.

**Supplementary Table 1 | Ionic conductivity of electrolytes.** The test temperature was about 25 ^o^C.

| **Electrolytes (0.25 M)** | **Conductivity (mS cm**^−^**^1^)** |
| --- | --- |
| BMIM-NTf_2_/PC | 4.25±0.05 |
| BMIM-PF_6_/PC | 4.65±0.04 |
| NBu_4_-NTf_2_/PC | 3.14±0.04 |
| NBu_4_-PF_6_/PC | 3.35±0.03 |
| SiO_2_-IL-NTf_2_/PC | 2.06±0.04 |
| SiO_2_-IL-PF_6_/PC | 2.34±0.04 |
| SiO_2_-IL-BMIM/PC | 1.53±0.05 |
| SiO_2_-IL-NBu_4_/PC | 1.04±0.06 |

**Supplementary Note 1**

Predictably, the grafted ions should be restricted to access the pores of the two ACs. But it is necessary to perform experiments to address the point whether the grafted ions do not contribute to charge storage. Thus we synthesized a material named as SiO_2_-MIM–SiO_2_-NTf in which both cations and anions were grafted to SiO_2_, and its chemical structure was confirmed by ^1^H NMR spectrum (Supplementary Figure 17). The SiO_2_-MIM–SiO_2_-NTf can be dissolved in PC solvent with a small solubility. Although it was hard to accurately determinate its ion concentration in PC because this material may have different solubility in PC and *d*-DMSO (*d*-DMSO was used as the solvent for ^1^H NMR experiment), a very dilute SiO_2_-MIM–SiO_2_-NTf/PC electrolyte was still obtained. As shown in Supplementary Figure 18, the cyclic voltammetry (CV) curve of YP-50F electrode in SiO_2_-MIM–SiO_2_-NTf/PC electrolyte exhibited a distorted rectangular shape which may be due to the slow mobility of the grafted ions. Comparison of this CV curve with that in BMIM-NTf_2_/PC electrolyte showed that the grafted ions led to a very low and negligible specific capacitance (Supplementary Figure 18). Here, it should be mentioned that the grafted ions led to significantly decreased but not zero specific capacitance because the grafted ions could be also adsorbed on the apparent electrode surface. Moreover, we can find that, the capacitance contributions by cations (BMIM^+^, NBu_4_^+^ shown in Figures 3a, c) at highly positively polarized potential and those by anions (NTf_2_^−^, PF_6_^−^ shown in Figure 3b, d) at highly negatively polarized potential were significantly decreased. So we defined the potential at which the maximum slope took place as the upper acting potential and the lower acting potential for cations and anions, respectively.

**Supplementary Note 2**

For EQCM experiment, a thin and homogenous sample-coating on quartz substrate is necessary to make sure that the additional mass has no effect on the acousto-elastic property of quartz^4,6,7^. With regard to the preparation of the YP-50F quartz crystal electrode, we used an organic binder (polyvinylidene fluoride (PVDF) dissolved in N-methyl-2-pyrrolidone (DMP)) instead of the previous aqueous binder (PTFE dissolved in water, used for the CV measurements). It is because that the gold-coated quartz crystal surface is hydrophobic, using aqueous PTFE binder makes it difficult to homogenously distribute carbon powder on gold-coated quartz crystal surface. Moreover, it has been demonstrated that a rigid electrode can be obtained when the carbon powder mixed with PVDF binder, which is the necessary condition for gravimetric application of EQCM^6,7^. Furthermore, it is important to make sure that the materials (i.e. carbon powder, PVDF and DMP) were strictly dried and the operation was carried out in a dry environment (in the oven, for example). It is because the presence of water could cause the aggregation of the binder, which is unfavorable to obtain a uniform coating. In our experiment, the slurry prepared by mixing 90 wt% carbon powder, 10 wt% PVDF in DMP and drops of DMP (the total [volume](javascript:void(0);) of DMP was about 80 µL with respect to 10 mg carbon powder) was [manual](javascript:void(0);)ly coated on the quartz crystal. Supplementary Figure 22 showed the SEM images of YP-50F coated on the quartz crystal surface.

**Supplementary Note 3**

The Sauerbrey’s Equation was used under the assumption that only capacitive behavior was involved on the electrode^4^. The operating potential window of the YP-50F-coated quartz electrode was smaller than that of the conventional electrodes because large potential window may result in non-capacitive behavior on the quartz electrode with very low loading of carbon^4,5^. The chronoamperometry test of the YP-50F-coated quartz electrode in 0.25 M BMIM-NTf_2_/PC electrolyte was performed to determine the operating potential window of EQCM experiment (Supplementary Figure 23)^4^. With the electrode negatively and positively polarized (Supplementary Figure 23a and 23b), the corresponding current response (Supplementary Figure 23c and 23d) and quartz resonance frequency (Supplementary Figure 23e and 23f) were recorded. To ensure that only capacitive behavior was involved on the electrode, the recorded current should be zero when the electrode polarized to a stable potential. As seen from Supplementary Figure 23c and 23d, the current could keep close to zero (the current value was defined less than 0.25 µA, Supplementary Figure 24) when the electrode polarized within −0.2 to 0.9 V/ref, but increased when the electrode polarized below −0.4 V/ref or above 1.1 V/ref. Correspondingly, the quartz resonance frequency could maintain a regular change from −0.2 to 0.9 V/ref (Supplementary Figure 23e, 23f). Therefore, the operating potential window in this work was selected to be −0.2 to 0.9 V/ref.

**Supplementary Note 4**

The application of EQCM for gravimetric mode implies that the mass change (Δ*m*) is linked exclusively to the resonant frequency change (Δ*f*) whereas the simultaneous resonance width change (Δ*W*) is zero or much smaller than the resonant frequency change (Δ*f*)^6,7^. The instrument we used, i.e. Princeton, QCM922A, can measure the resonance resistance change (Δ*R*), which is transformed into the related resonance width change (Δ*W*) by Supplementary Equation (1)^8^:

$\text{Δ}W=\frac{16Ae_{26}^{2}\rho_{q}f_{q}^{3}}{\pi Z_{q}^{3}}\cdot\text{Δ}R$ (1)

where *e*_26_ is the piezoelectric stress coefficient (9.65 × 10^−2^ C m^−2^), *Z_q_* is the acoustic wave impedance (8.8 × 10^6^ kg m^−2^ s^−1^). Thus the resonance width change (Δ*W*) can be obtained by multiplying the resonance resistance by a factor of 14. In our system, the resonance resistance change was about 6 Ω when the YP-50F-coated quartz electrode was cycled between −0.2 V to 0.9 V (Supplementary Figure 25a). The resonant frequency change (Δ*f*) overwhelmingly prevailed over the resonance width changes (Δ*W*), indicating a gravimetric behavior of the quartz-crystal microbalance (Supplementary Figure 25b).

**Supplementary Methods**

**Preparation and characterization of porous activated carbons (ACs)**

The commercial available AC YP-50F (Kuraray Chemical, Japan) was washed with deionized water and annealed at 700 ^o^C under argon to remove impurities prior to use. The specific surface area is 1533 m^2^ g^−1^ (Supplementary Figure 1).

Sodium alginate-derived AC (SADC) was synthesized as follows: Sodium alginate was firstly pyrolyzed at 400 ^o^C under argon atmosphere for 2 h to obtain pre-carbonization product (PCSA). For the subsequent NaOH activation: typically, 1.0g PCSA was impregnated with 2.0 g NaOH in aqueous solution under strong agitation followed by evaporation at 80 ^o^C under vacuum. The PCSA/NaOH mixture was heated at 800 ^o^C for 1 h under argon atmosphere. After being cooled down to room temperature, the product was washed with HCl (1M) and deionized water, then dried at 60 ^o^C in air. The specific surface area of SADC is 2244 m^2^ g^−1^ (Supplementary Figure 2).

**Determination of ion concentrations of silica nanoparticles-grafted ionic liquids**

The ion concentrations of SiO_2_-grafted ILs were determined by an internal standard method. In a typical procedure, mesitylene (10 µL, 0.0721 mmol), which acted as internal standard, was added to a solution of SiO_2_-grafted IL (50 mg) in *d*-DMSO. The ^1^H NMR spectrum of this solution was recorded (Supplementary Figures 9–12). The ion concentration (*n*, (mmol g^−1^)) of the SiO_2_-grafted IL was calculated by comparing the relative peak areas of mesitylene and SiO_2_-grafted IL (Supplementary Equation 2):

$n=\frac{A/N}{A_{o}/N_{o}}\cdot\frac{0.0721}{0.05}$ (2)

where *A* is the relative peak area and *N* is the number of corresponding hydrogen atoms of SiO_2_-grafted IL. *A_o_* is the relative peak area and *N_o_* is the number of corresponding hydrogen atoms of mesitylene.

After the ion concentrations were determined, the SiO_2_-grafted ILs were dissolved in propylene carbonate (PC) with the same calculated concentration of 0.25 M.

**Inductively coupled plasma optical emission spectroscopy**

The synthesized SiO_2_-grafted ILs were digested with hydrofluoric acid (HF). After removing HF by heating, the amount of Li was measured by inductively coupled plasma optical emission spectroscopy (ICP–OES). The results showed that the absence of Li in each SiO_2_-grafted IL.

**Electrochemical characterization**

BMIM-NTf_2_, BMIM-PF_6_, NBu_4_-NTf_2_ and NBu_4_-PF_6_ ILs were purchased from Center for Green Chemistry and Catalysis, Lanzhou Institute of Chemical Physics, Chinese Academy of Sciences and vacuum-dried before use. All of the purchased ILs and the synthesized SiO_2_-grafted ILs were dissolved in anhydrous propylene carbonate (PC, Sigma-Aldrich) as electrolytes with the same concentration of 0.25 M. Taking BMIM-NTf_2_/PC as an example, as shown in Supplementary Figure 16, the concentration in the range of 0.125 M to 1.00 M has little effect on the capacitance of YP-50F electrode. Moreover, higher concentration would lead to higher viscosity and/or poorer solubility for SiO_2_-grafted ILs. Thus, we chose the concentration of 0.25 M in this study.

**Electrochemical quartz crystal microbalance (EQCM) experiment**

Before EQCM measurement, the potential of zero charge (pzc) for the system of YP-50F in BMIM-NTf_2_ was determined by CV tests using a closed three-electrode system^4^: a YP-50F electrode with a mass loading of 2 mg was used as the working electrode, another YP-50F electrode with a mass loading of 10 mg was used as the counter electrode, and a silver disk electrode was used as the quasi-reference electrode. The YP-50F electrodes were prepared as described before. Supplementary Figure 21 showed the CV curves of the YP-50F working electrode tested in BMIM-NTf_2_/PC electrolytes with different concentrations of 1.00, 0.50 and 0.25 M at a scan rate of 5 mV s^−1^. The pzc, defined at minimum capacitance (dC/dE = 0), was about 0.27 V/ref. Moreover, the measured open circuit potential was about 0.21 V/ref.

EQCM can measure the resonance frequency change (Δ*f*), which can be converted to the mass change (Δ*m*) by Sauerbrey’s Equation (3):

$\Delta m=-\frac{A\sqrt{\mu_{q}\rho_{q}}}{2f_{q}^{2}}\cdot\Delta f=-C_{f}\cdot\Delta f$ (3)

where *A* is the area of active surface (0.198 cm^2^), *µ_q_* is the AT-cut quartz constant (2.947 × 10^11^ g cm^−1^ s^−2^), *ρ_q_* is the quartz crystal density (2.65 g cm^−3^), *f_q_* is the reference frequency (9.00 MHz). Here, the sensitivity factor *C_f_* is 5.682 ng Hz^−1^ cm^−2^.

The experimental ion population change (Δ*Γ*) was calculated by Supplementary Equation (4):

$\Delta\Gamma=\frac{\Delta m}{Mi}$ (4)

where Δ*m* is the mass change, *Mi* is the molecular mass, and for ΔQ < 0, *Mi* = *M*(BMIM^+^) = 139.1 (g mol^−1^); for ΔQ > 0, *Mi* = *M*(NTf_2_^−^) = 279.9 (g mol^−1^).

The theoretical ion population change (Δ*Γ_theor_*) was calculated by using Faraday’s law, i.e. Supplementary Equation (5):

$\Delta\Gamma_{theor}=\frac{\Delta Q}{nF}$ (5)

where Δ*Q* is the charge passed through the electrode (*C*), *n* is the valence number of the ion (*n* = 1), and *F* is the Faraday constant (96485 C mol^−1^).

**Supplementary References**

1. Lu, Y., Moganty, S. S., Schaefer, J. L. & Archer, L. A. Ionic liquid-nanoparticle hybrid electrolytes. *J. Mater. Chem*. **22**, 4066–4072, (2012).
2. Dillon, R. E. A., Stern, C. L. & Shriver, D. F. Influence of the anion on the formation of amorphous ionically conducting lithium salt complexes with 18-C-6 and 2.2.2-cryptand macrocycles. *Chem. Mater.* **13**, 2516–2522, (2001).
3. Paschoal, V. H., Faria, L. F. O. & Ribeiro, M. C. C. Vibrational spectroscopy of ionic liquids. *Chem. Rev.* **117**, 7053–7112, (2017).
4. Griffin, J. M. et al. In situ NMR and electrochemical quartz crystal microbalance techniques reveal the structure of the electrical double layer in supercapacitors. *Nat Mater.* **14**, 812–819, (2015).
5. Tsai, W.Y., Taberna, P.L. & Simon, P. Electrochemical quartz crystal microbalance (EQCM) study of ion dynamics in nanoporous carbons. *J. Am. Chem. Soc.* **136**, 8722–8728, (2014).
6. Levi, M.D., Sigalov, S., Aurbach, D. & Daikhin, L. In situ electrochemical quartz crystal admittance methodology for tracking compositional and mechanical changes in porous carbon electrodes. *J. Phys. Chem. C* **117**, 14876–14889 (2013).
7. Levi, M.D. et al. In situ porous structure characterization of electrodes for energy storage and conversion by EQCM-D: a review. *Electrochim. Acta*. **232**, 271–284 (2017).
8. Johannsmann, D. The quartz crystal microbalance in soft matter research. (Springer, 2015).
